# Supplementary figures and images for: Experimental transmission of Leishmania (Mundinia) parasites by biting midges (Diptera: Ceratopogonidae)
Source: PLoS Pathog. 2021 Jun 11;17(6):e1009654. doi: 10.1371/journal.ppat.1009654 (PMC8221790; doi:10.1371/journal.ppat.1009654)

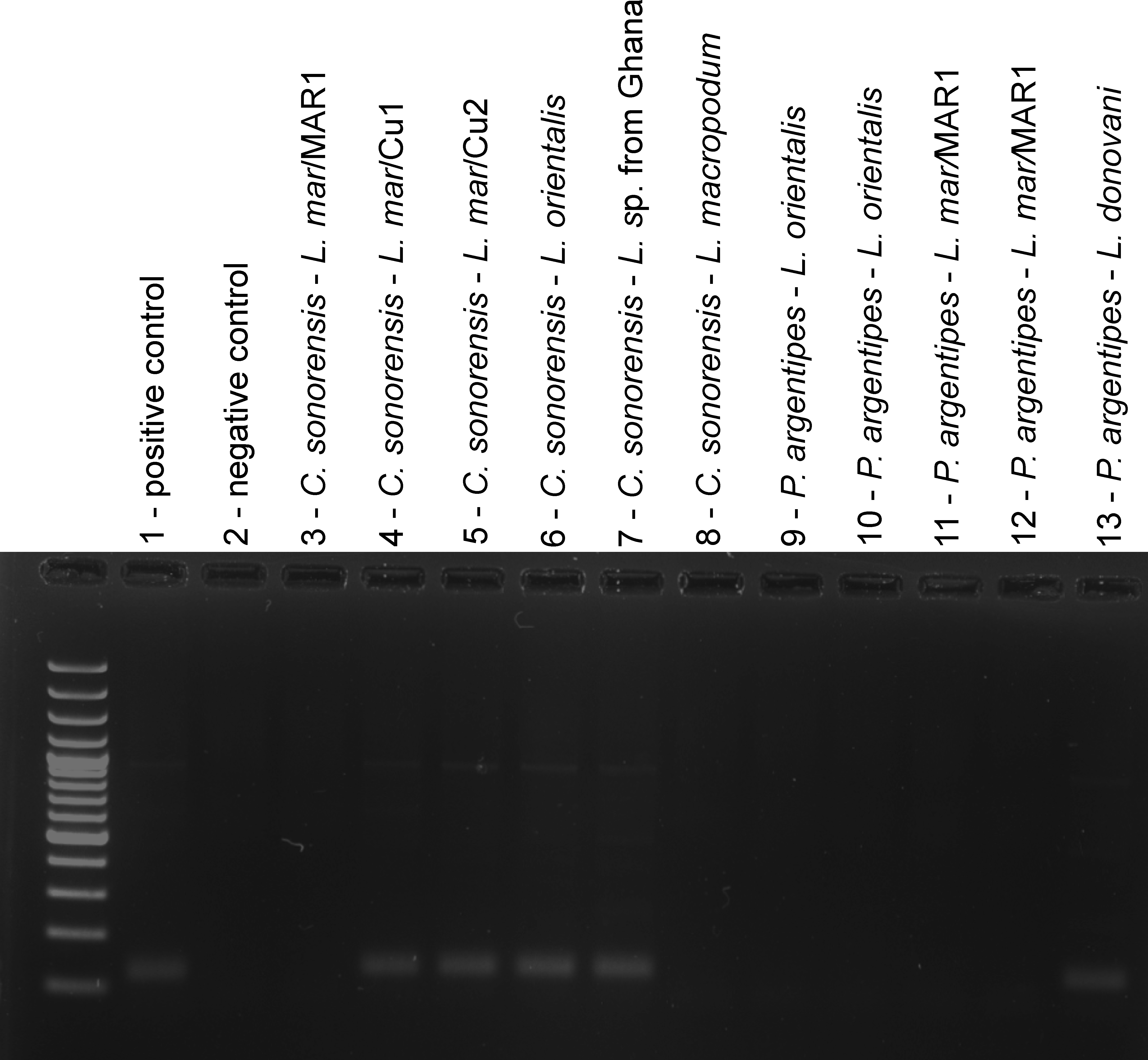

Supplement: S1 Fig — 1, Positive control from cultured parasites; 2, Negative control; 3–8, Mouse ear exposed to biting midges (Culicoides sonorensis) infected with L. martiniquensis Mar1 (3), L. martiniquensis Cu1 (4), L. martiniquensis Cu2 (5), L. orientalis (6), L. sp. from Ghana (7), L. macropodum (8); 9–13, Mouse ear exposed to P. argentipes infected with L. orientalis (9, 10), L. martiniquensis Mar1 (11, 12) and L. donovani (13). (TIF) [file ppat.1009654.s001.tif]
