# Supplementary material for: Experimental transmission of Leishmania (Mundinia) parasites by biting midges (Diptera: Ceratopogonidae)
Source: PLoS Pathog. 2021 Jun 11;17(6):e1009654. doi: 10.1371/journal.ppat.1009654 (PMC8221790; doi:10.1371/journal.ppat.1009654)
Supplement: Attachment — Submitted filename: response to reviewers.docx [file ppat.1009654.s002.docx]

**RESPONSE TO REVIEWERS**

We are grateful to both referees for all their comments and valuable suggestions.

The detailed list of our responses follows:

**Major Issues: Key Experiments Required for Acceptance**

*Reviewer #1: The weakest point of the paper involves the apparent absence of successful transmission of Mundinia sp. by P. argentipes. While this is formally true based on the limited data, the evidence is weak. The possible transmission of L. orientalis by P. argentipes might be expected given the presence of mature infections in 20% of the flies. However, the transmission experience they describe is limited to a single experiment in which only 2 infected, engorged flies were observed, along with 5 unfed flies that showed colonization of the stomodeal valve. More critically, the positive control showing that the colonized P. argentipes flies can transmit L. donovani was not included. By contrast, this control involving P. argentipes infected with L. donovani was included in the comparisons of parasite development in the midgut (fig 2). Since the transmission experiments provide the principle advance of this paper, a more complete study should be shown. Furthermore, the ability of P. argentipes to transmit L. orientalis is itself a biologically relevant question given the sympatric distribution of these sand fly and parasite species in southeast Asia.*

According to the reviewer request, we performed additional transmission experiments with *P. argentipes* infected with *L. donovani* (control) and two *Mundinia* species - *L. orientalis* (20% mature infections) and *L. martiniquensis* MAR1 (7% mature infections). Transmission was achieved with *L. donovani* (Table 3, Fig S1). *Mundinia* species were not transmitted, recapitulating the first experiment, despite the numbers of infected females feeding on mice being high. We believe that the negative results are now better supported. We completed these data to Table 3, Fig S1 and to the text on the lines 266 and 279-280.

*Reviewer #2: I have no major problems/issues with this manuscript. However, the “Metacyclic Forms” section in Results requires additional data/information as to how metacyclic forms were identified, as well as how they differ from procyclic forms. The single parasites in Figure 4 panels C and D alone are not sufficient.*

A thorough description of *Mundinia* morphological forms present in vectors (using fluorescent and electron microscopy) is our next goal, intended for a separate paper. However, we understand the request to provide more information on this topic. The method of identification of metacyclic forms was described in details (lines 432-437) and more images showing the spectrum of procyclic forms were added to the Figure 6 (figure caption on lines 237-243).

***Minor Issues: Editorial and Data Presentation Modifications***

*Reviewer #1: No statistical comparisons of the infection parameters were performed, either between development of the same parasite in midges vs. sand flies, or between different parasite strains in the same vector.*

We performed Chi-square tests to compare intensities of infections. The statistics for differences between *Leishmania* species in the same vector were added to the captions of the Fig 2 (lines 156-158), Fig 3 (lines 171-173), Fig 4 (lines 195-198) and Fig 5 (lines 234-236). The statistics for differences between the development in midges vs. sand flies were added to Table 1 (lines 226-227). One sentence with description of the method and specification of statistical software was added to Methods, lines 430-431.

*The highest representation of metacyclics in the infected midges was 5 % and 10 %. These are very low frequencies in comparison to the frequencies (>50%) found in sand flies transmitting Leishmania sp. The authors might comment on this point in the discussion.*
We added a paragraph on representation of metacyclics (citing three new references) to discussion (lines 382-392).

*For the insect colonies used, specify the geographic origin of the wild caught flies used to establish the colony.*

The geographic origin was specified (lines 406-408).

*Reviewer #2: INTRODUCTION, Lines 66-67: what is meant by “via”? This word infers a logical progression from cutaneous to diffuse cutaneous and mucocutaneous to visceral lesihmaniasis. Is this true?*

The word “via” was misleading; we deleted it (lines 66-67).

*Line 107: The “G” in guinea pig should be lower-case.*

Corrected (line 107)

*Lines 113 - 115: Say that L. macro was detected in biting midges collected during circulation of disease in red kanagaroos, but Line 100 says there are no reports of CL in wild populations; very confusing as written.*

We changed the sentence on line 114 from „…collected during circulation of disease in red kangaroos” to „…in areas of Australian *Leishmania* transmission“

*Line 120: “…met three from four Killick-Kendrick’s criteria…”. Maybe replace “from” with “of the”?*

Replaced (line 119)

*RESULTS, Line 143-153: Data showing the failure/poor development of L. Mundinia species in L. migonei is important, and should be included in the body of the paper (not as Supplementary Figure S1).*

The figure was included in the body of the paper as Figure 2.

*Line 154-160: Data showing the failure of L. Mundinia species to develop in P. duboscqi is important, and should be included in the body of the paper (not as Supplementary Figure S2). Line 159, might help to ass in the word “control” before “L. major”.*

The figure was included in the body of the paper as Figure 3. The word “control” was added, line 164.

*Line 179: Need to define/identify what “LSCM$” and “CUK3” are. I could not find them in Fig. 2.*

The labels LSCM4 and CUK3 were redundant here and were deleted.

*Line 187-188: Why isn’t Day 1 data presented? How many females were infected on Day 1 post-blood meal? How “heavy” were these infections? Not sufficient to only state all parasites were still enclosed in the blood meal.*

On day 1 PBM, we performed only control dissections to confirm that leishmania are viable (3 females per strain) and keep more material for following days. Infection rates were 100% and heavy infections were present in all cases. We completed this information into the text, lines 202-203.

*Line 197-199: What happened to L. enrietti parasites on Day 6 PBM (Fig. 3 panels C,D)?*

*L. enriettii* was affected with defecation of bloodmeal remnants in high proportion of vectors. They survived in a small part of females, not revealed on day 6 but later on day 10 PBM. We added this information to the text on lines 215-217.

*Line 203-205: Why was GHA species omitted from this statement? Looks to me that GHA established a heavy infection and successful colonization of the SV, similar to ORI and MAR-Cu2.*

The reviewer is right; we included GHA species to the sentence (line 223)

*Line 215-217: It is very difficult to see the parasites in Figure 4 panel A. An arrow pointing to the parasites, or a dotted line around the parasites, would be very helpful. Easier to see parasites in panel B, but would not hurt to clearly identify the parasites in this panel as well.*

We modified the Figure 4 (now Figure 6) and marked the part of the thoracic midgut filled with parasites by a red dotted line.

*Line 219-235: This entire section is lacking crucial information about the criteria used to identify “metacyclic forms”. Furthermore, would be informative to show side-by-side pictures of procyclic and metacyclic forms (rather than the single parasites currently in Figure 4 panels C and D). In Lines 232-233, would be helpful to include the Figure where this data was presented: “…(L. orientalis and L. martiniquensis MAR 1; Fig. 2).”*
The method of identification of metacyclic forms was completed (lines 432-437) and more images showing the shape spectrum of procyclic forms were added to the Figure 6 (figure caption on lines 237-243, text on lines 253-254). The reference to Fig 6C, D was moved to the line 252.

*DISCUSSION, Lines 326 -327: Why do the authors think that geographically distant isolates of L. MAR developed better in P. argentipes than sympatric isolates?*

Our hypothesis is that MAR1 strain might be better adapted to sand flies while Cu1 and Cu2 strains to biting midges. This hypothesis needs further investigations, however, and we omitted to reduce speculation in discussion.
